# Variable-Amplitude Fatigue Life Prediction of SBS-Modified Asphalt Mixtures with Physical Gel Structures

**DOI:** 10.3390/gels12090825

**Published:** 2026-09-08

**Authors:** Chenze Fang, Jiahao Yang, Menghao Wang, Hongbin Zhu, Pingfan Hu

**Affiliations:** 1 School of Civil Engineering and Transportation, North China University of Water Resources and Electric Power, Zhengzhou 450045, China; fangchenze@ncwu.edu.cn (C.F.); 15639598669@163.com (J.Y.); 2 School of Civil Engineering, Suzhou University of Science and Technology, Suzhou 215009, China; 3 College of Transportation Engineering, Dalian Maritime University, Dalian 116026, China; 4 Artie McFerrin Department of Chemical Engineering, Texas A&M University, College Station, TX 77843-3122, USA; pingfanhu@tamu.edu

**Keywords:** road engineering, asphalt mixture, nonlinear damage accumulation, loading sequence–interaction, fatigue life prediction

## Abstract

The service life of SBS-modified asphalt mixtures with physical gel structures is strongly influenced by the loading sequence–interaction coupling effects. However, conventional linear models fail to accurately capture the influence of such coupling effects on fatigue life. This study, therefore, aims to develop a life prediction method for SBS-modified asphalt mixtures that explicitly accounts for the loading sequence–interaction coupling effects. First, indirect tensile monotonic loading tests, as well as constant-amplitude and variable-amplitude indirect tensile repeated loading tests were conducted to determine the fatigue life of SBS-modified asphalt mixtures under different loading modes. Subsequently, a viscoelastic fatigue damage model was developed to analyze the accumulation of fatigue damage in SBS-modified asphalt mixtures. Subsequently, the effects of loading sequence and loading interaction on the fatigue damage accumulation process in SBS-modified asphalt mixtures were investigated. Based on these analyses, a nonlinear fatigue damage accumulation model considering the loading sequence–interaction coupling effects was established. Finally, a nonlinear fatigue damage accumulation factor was introduced for developing a fatigue life prediction model for SBS-modified asphalt mixtures considering the loading sequence–interaction coupling effects. The results indicate that the low–high (*σ*_low_–*σ*_high_) and high–low (*σ*_high_–*σ*_low_) loading sequences, respectively, retard and accelerate the fatigue damage accumulation process of SBS-modified asphalt mixtures. Consequently, the corresponding cumulative fatigue life fractions are greater than 1 and less than 1, respectively. Furthermore, the resulting fatigue life is positively correlated with the first-level fatigue life fraction under the *σ*_low_–*σ*_high_ sequence but negatively correlated under the *σ*_high_–*σ*_low_ sequence. The developed nonlinear fatigue damage accumulation model can accurately track the nonlinear fatigue damage accumulation of SBS-modified asphalt mixtures. The established nonlinear fatigue life prediction model accurately predicts the fatigue life of SBS-modified asphalt mixtures with physical gel structures under cyclic loading with variable stress amplitudes.

## 1. Introduction

Styrene–butadiene–styrene (SBS)-modified asphalt is a typical viscoelastic material with physical gel structures and is the primary paving material for high-grade pavements. During its service life, it is subjected to long-term cyclic loading from vehicles with varying loading amplitudes. Fatigue cracking induced by such loading is a major source of distress that threatens the durability of pavement structures. Reordering different loading amplitudes can lead to variations in fatigue life. This phenomenon is known as the loading sequence effect [1,2]. Nevertheless, the commonly employed Miner’s linear damage accumulation rule [3] postulates that fatigue damage is independent of loading sequence. For this reason, this rule cannot properly describe the nonlinear damage accumulation characteristics of asphalt mixtures subjected to variable-amplitude loading. Reliable estimation of variable-amplitude fatigue life for SBS-modified asphalt mixtures carries important scientific implications for improving fatigue design of SBS-modified asphalt pavements and prolonging their service life [4].

Actual vehicle loading exhibits significant variable-amplitude characteristics due to variations in vehicle type, axle weight, and other factors [5]. Consequently, the fatigue response of SBS-modified asphalt pavements under such loading differs considerably from that under constant-amplitude loading conditions. The conventional Miner’s linear accumulation rule ignores the effect of loading sequence on the damage accumulation process of materials. This leads to considerable discrepancies between predicted and actual fatigue lives under variable-amplitude loading [6,7,8]. To address this issue, extensive research has been conducted on metallic materials by scholars worldwide. Marco and Starkey [9] were among the first to propose a theoretical framework for nonlinear fatigue damage accumulation and applied it to the study of crack propagation laws. Ye and Wang [10] established a nonlinear accumulation model based on toughness degradation. Mesmacque et al. [11] and Kwofie et al. [12] revealed the mechanisms by which loading sequence affects damage accumulation, based on the damage stress increment and the fatigue driving stress, respectively. The aforementioned findings have laid an important theoretical foundation for analyzing the effect of loading sequence on SBS-modified asphalt materials. However, as a typical viscoelastic material, SBS-modified asphalt with physical gel structures exhibits fatigue damage evolution that is strongly dependent on temperature, loading frequency, and time [13,14,15]. Therefore, the damage theories developed for metallic materials cannot be directly applied to characterize the damage behavior of SBS-modified asphalt materials [16,17]. There is an urgent need to establish a variable-amplitude fatigue life prediction method specifically for SBS-modified asphalt pavement materials.

In recent years, increasing research efforts have been devoted to exploring loading sequence effects on the fatigue performance of asphalt mixtures [18,19]. Fang et al. [20,21,22] constructed a nonlinear fatigue damage accumulation model for asphalt materials, which takes loading sequence effects into consideration, on the basis of stress-controlled constant-amplitude and variable-amplitude indirect tensile cyclic loading tests. Wang et al. [23] carried out variable-amplitude four-point bending fatigue experiments to investigate loading sequence impacts on the stiffness modulus degradation of asphalt mixtures. Jiang et al. [24] proposed a variable-amplitude cyclic loading test protocol to examine loading- sequence-induced variations in the permanent deformation of asphalt mixtures. Yao et al. [25] applied machine-learning-based methods to evaluate how loading-amplitude order governs the cracking resistance of asphalt pavements. The above-mentioned works provide critical theoretical underpinnings for interpreting the fatigue responses of SBS-modified asphalt mixtures under variable-amplitude loading.

To date, several fatigue damage models have been developed for asphalt materials. Li et al. [13,14] investigated the kinetics-based fatigue crack growth and stochastic damage behavior in viscoelastic materials, highlighting the rate-dependent nature of damage evolution. Zhang and Oeser [19] further revealed the energy dissipation characteristics and rheological property degradation of asphalt binders under repeated shearing. Fang et al. [26] extended nonlinear damage accumulation theory to asphalt binders by explicitly considering loading amplitude sequence and interaction effects. Despite these advances, the coupled influences of loading sequence and loading interaction on the fatigue damage accumulation of SBS-modified asphalt mixtures with physical gel structures remain insufficiently addressed, particularly under variable-amplitude indirect tensile loading conditions. Even so, although several previous studies covered SBS-modified asphalt mixtures, these materials were only adopted for model validation. There remains a scarcity of systematic mechanical characterizations focusing specifically on the nonlinear damage accumulation features of SBS-modified asphalt mixtures subjected to variable-amplitude loading.

The loading history generated by one preceding cycle within the axle-loading spectrum may alter fatigue damage buildup for the succeeding cycle. This phenomenon is termed the loading interaction effect [27,28]. Most existing investigations concentrate on describing loading sequence impacts on damage accumulation of asphalt materials, yet they fail to fully consider the mechanical influences of loading interactions on fatigue damage buildup within SBS-modified asphalt mixtures. Throughout pavement service periods, SBS-modified asphalt mixtures undergo sophisticated nonlinear damage evolution under the combined actions of loading sequence and loading interaction [29,30]. Nevertheless, the mechanical mechanism governing how the coupling between loading sequence and loading interaction influences nonlinear damage accumulation, as well as the variable-amplitude fatigue life of SBS-modified asphalt mixtures, is still lacking systematic clarification. Accordingly, relevant theoretical frameworks for service life prediction are yet to be developed. The three-dimensional physical gel network and entanglement structure of SBS-modified asphalt have been extensively documented and are generally accepted in the pavement engineering community [15]. Accordingly, this study concentrates on the macroscopic mechanical behavior and fatigue damage evolution, without undertaking additional microstructural characterization.

Given these concerns, the present study focuses on SBS-modified asphalt mixtures. A series of tests was conducted at 20 °C and 10 Hz, including indirect tensile monotonic loading tests and constant- and variable-amplitude indirect tensile repeated loading tests, to obtain fatigue life results under different loading modes. A viscoelastic fatigue damage model was then developed to analyze the accumulation of fatigue damage in SBS-modified asphalt mixtures. Subsequently, the mechanisms by which loading sequence and loading interaction influence the damage accumulation process were investigated, based on which a nonlinear fatigue damage accumulation model considering their coupling effects was established. Finally, a nonlinear fatigue damage accumulation factor was introduced to modify the conventional linear fatigue equation, leading to the development of a variable-amplitude fatigue life prediction model for SBS-modified asphalt mixtures. This model provides a theoretical basis for the life prediction of SBS-modified asphalt mixtures under variable-amplitude loading.

## 2. Results and Discussion

### 2.1. Fatigue Damage Accumulation Analysis of SBS-Modified Asphalt Mixtures with Physical Gel Structures Based on Viscoelastic Damage Theory

#### 2.1.1. Damage Evolution Analysis Based on Viscoelastic Damage Theory

The Burgers model consists of a dashpot (*η*_0_) connected in series with a three-element Van der Poel model. The various strain components of SBS-modified asphalt mixtures with physical gel structures can be captured by the Burgers model with reasonable accuracy. By applying the binary-function correction shown in Equation (1) to the series dashpot, a permanent strain model (*ε_p_*, *N*) that excludes damage effects can be derived [31,32], as presented in Equation (2):

(1) $$\eta_{0}(t)=\frac{\eta_{0}}{at^{2}+bt+1}$$

where *a* and *b* are constants, with *a* > 0, *b* < 0, and *b^2^ −* 4*a* < 0; and *η*_0_ is the initial viscosity.

(2) $$\varepsilon_{p,N}=\alpha N^{3}+\rho N^{2}+\gamma N+\lambda (1-e^{-\kappa N})$$

The above model can depict the permanent strain response of asphalt mixtures within their undamaged state. Nevertheless, this model ignores the influences exerted by fatigue damage upon permanent strain. For this reason, it is essential to establish a permanent strain model for asphalt mixtures that takes damage-related effects into account [33,34,35,36,37]. Previous studies [6,27] have demonstrated that the damage evolution formulation defined by dissipated energy can reliably describe the progression of fatigue damage in viscoelastic materials, and the corresponding viscoelastic fatigue damage model expressed in Equation (3) is able to precisely capture the evolution process of fatigue damage in asphalt mixtures.

(3) $$D=1\;-{\left(1-\;\frac{N}{N_{f}}\right)}^{\frac{1}{1+2\beta }}$$

By combining Equations (2) and (3), the permanent strain model considering damage is derived as follows:

(4) $$\varepsilon_{p,N}=\frac{\varepsilon_{p,N}}{1-D}=\frac{\alpha N^{3}+\rho N^{2}+\gamma N+\lambda (1-e^{-\kappa N})}{{\left(1-\frac{N}{N_{f}}\right)}^{\frac{1}{1+2\beta }}}$$

**Figure 1:**
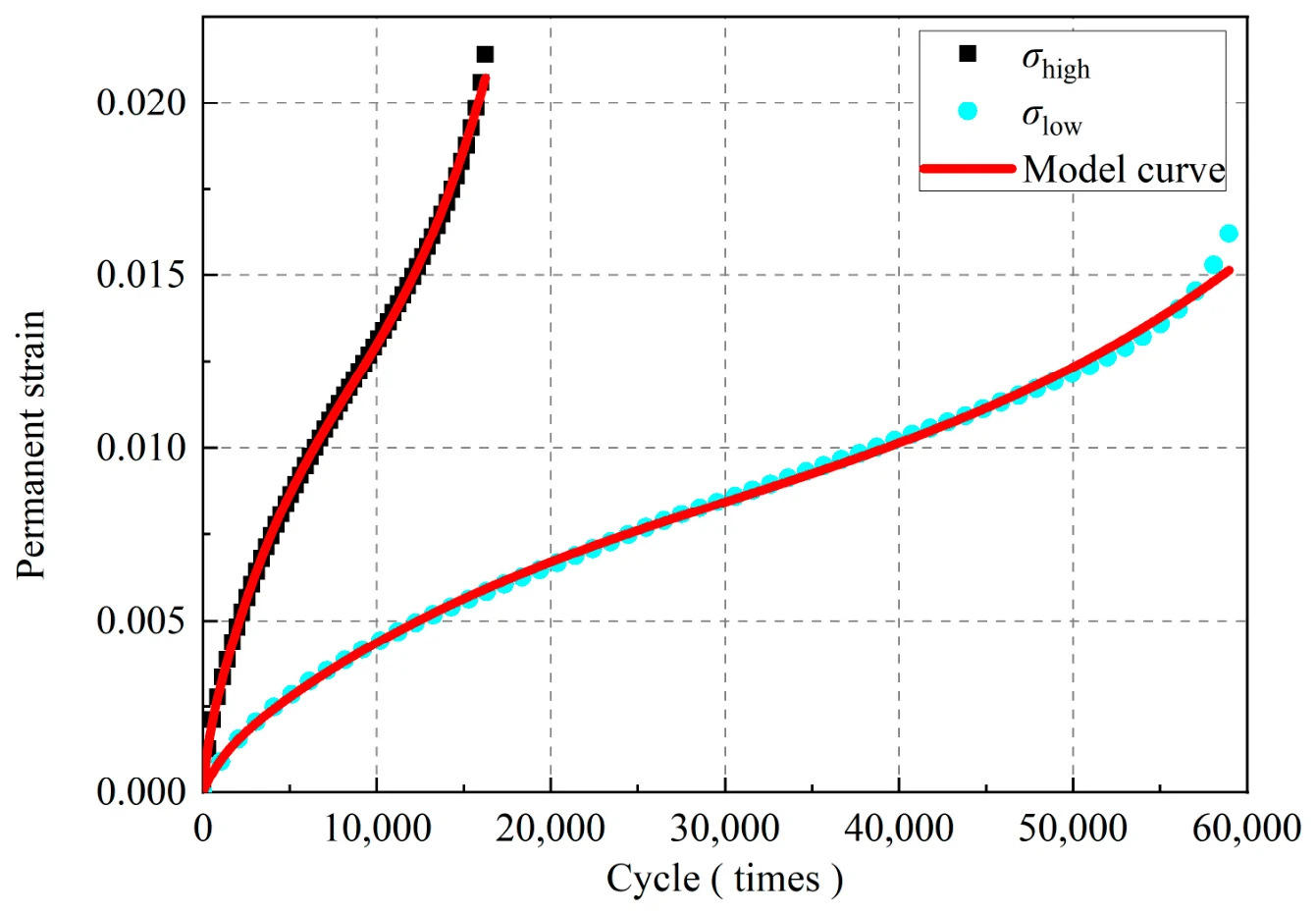
Fitting results of permanent strain model for SBS-modified asphalt mixtures.

**Figure 2:**
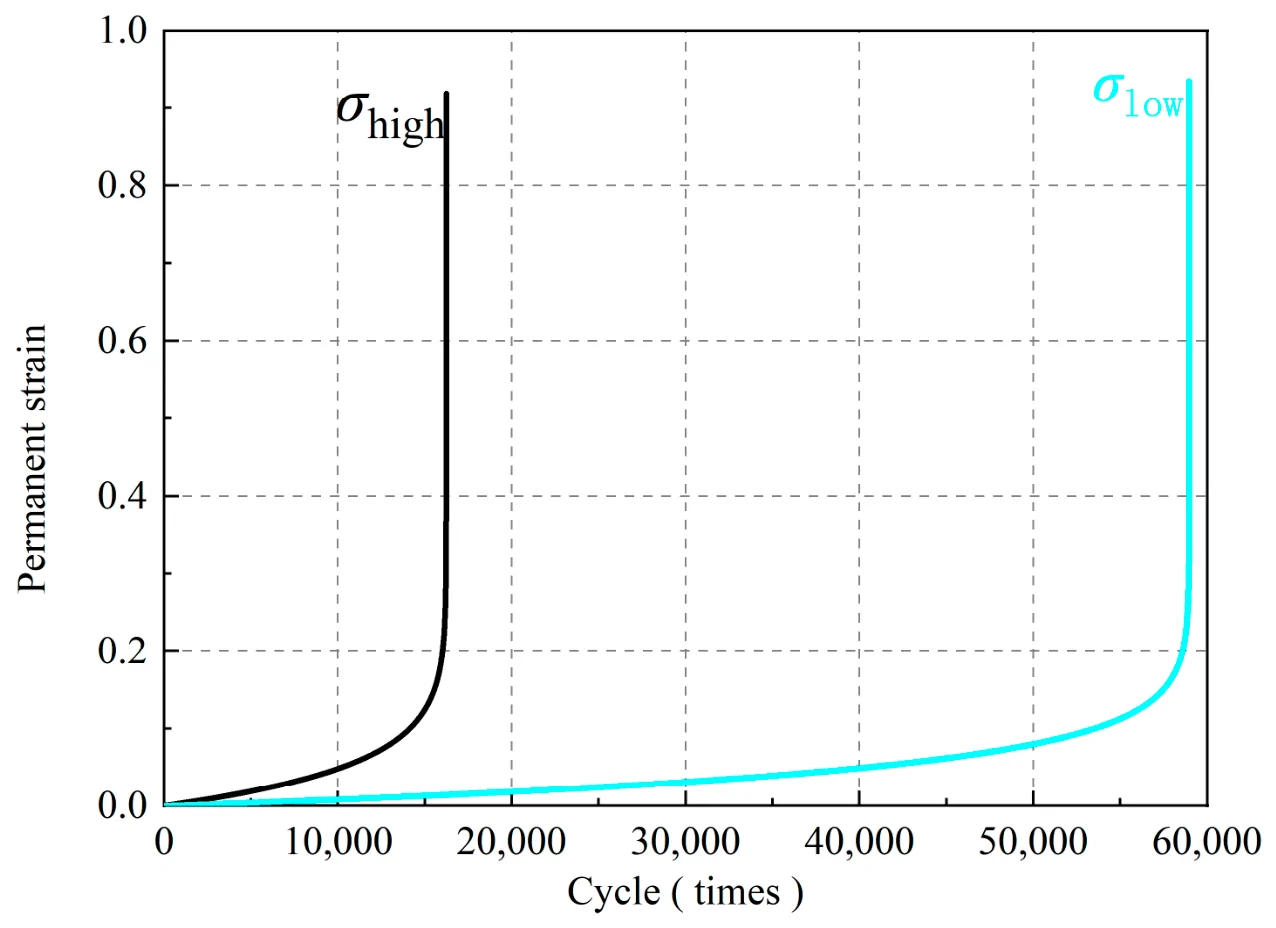
Fatigue damage curves of SBS-modified asphalt mixtures under different load levels.

The established permanent strain model, which accounts for damage, was used to fit the permanent strain curves at different load levels. The fitting results are shown in Figure 1 and Table 1. It can be seen from Figure 1 and Table 1 that the established permanent strain model considering damage can accurately describe the nonlinear evolution of permanent strain in SBS-modified asphalt mixtures during damage evolution. Furthermore, the obtained *β* was substituted into $D=1\;-{\left(1-\;\frac{N}{N_{f}}\right)}^{\frac{1}{1+2\beta }}$ to obtain the damage curves under different load levels, as shown in Figure 2. It can be seen from Figure 2 that the fatigue damage of SBS-modified asphalt mixtures at constant-amplitude indirect repeated tensile loading exhibits a nonlinear evolution trend. When the load amplitude decreases from *σ*_high_ to *σ*_low_, the slope of the damage curve decreases significantly, indicating that the damage evolution rate of SBS-modified asphalt mixtures is strongly dependent on the load amplitude and positively correlated with it.

#### 2.1.2. Analysis of Fatigue Damage Accumulation Under Repeated Indirect Tensile Loading with Constant Amplitude

**Figure 3:**
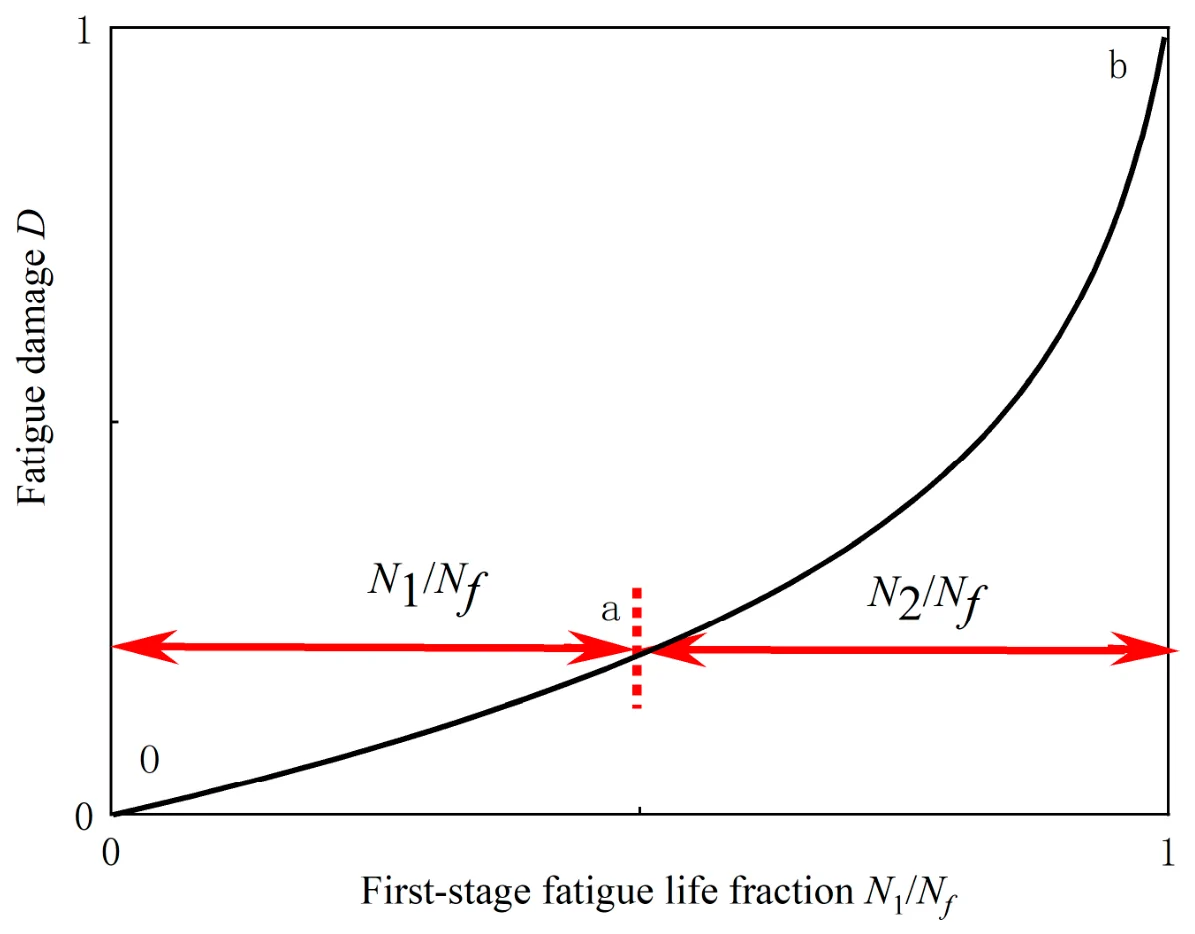
Schematic diagram of fatigue damage accumulation paths under constant stress amplitude cyclic loading.

This work investigates the fatigue damage evolution of SBS-modified asphalt mixtures under constant-amplitude cyclic indirect tensile loading, as illustrated in Figure 3. According to Figure 3, oa represents the damage accumulation path corresponding to *N*_1_ loading cycles applied at stress level *σ*, while ab stands for the succeeding path generated by *N*_2_ cycles under the same stress condition. For path oa, the first fatigue life fraction is *N*_1_/*N_f_*; for path ab, the second-stage fraction is *N*_2_/*N_f_*. Accordingly, the total cumulative fatigue life fraction for the entire accumulation process oa-ab is *N*_1_/*N_f_* + *N*_2_/*N_f_*.

**Figure 4:**
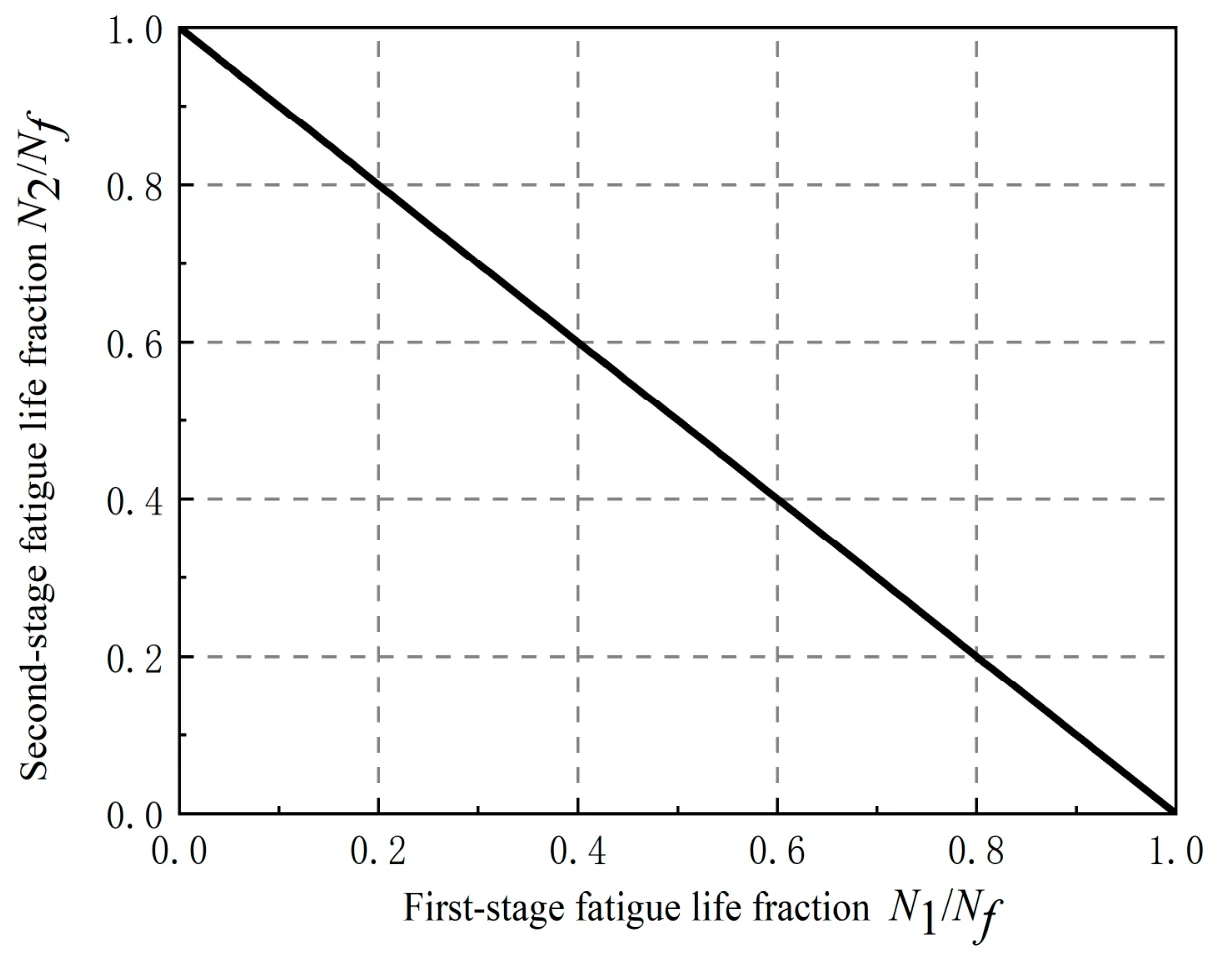
Second life fractions under constant stress amplitude cyclic loading.

Figure 4 presents the variation of *N*_2_/*N_f_* under the two-stage loading sequence. As *N*_1_/*N_f_* increases gradually from 0 to 1, *N*_2_/*N_f_* decreases linearly from 1 to 0. Moreover, this sum remains equal to 1 throughout, which is consistent with the cumulative fatigue life fraction predicted by Miner’s rule (*N*_1_/*N_f_* + *N*_2_/*N_f_* = 1). These results indicate that, although the fatigue damage in SBS-modified asphalt mixtures evolves nonlinearly under constant-amplitude repeated indirect tensile loading, the overall accumulation process is linear.

#### 2.1.3. Analysis of Fatigue Damage Accumulation Under Variable-Amplitude Repeated Indirect Tensile Loading

**Figure 5:**
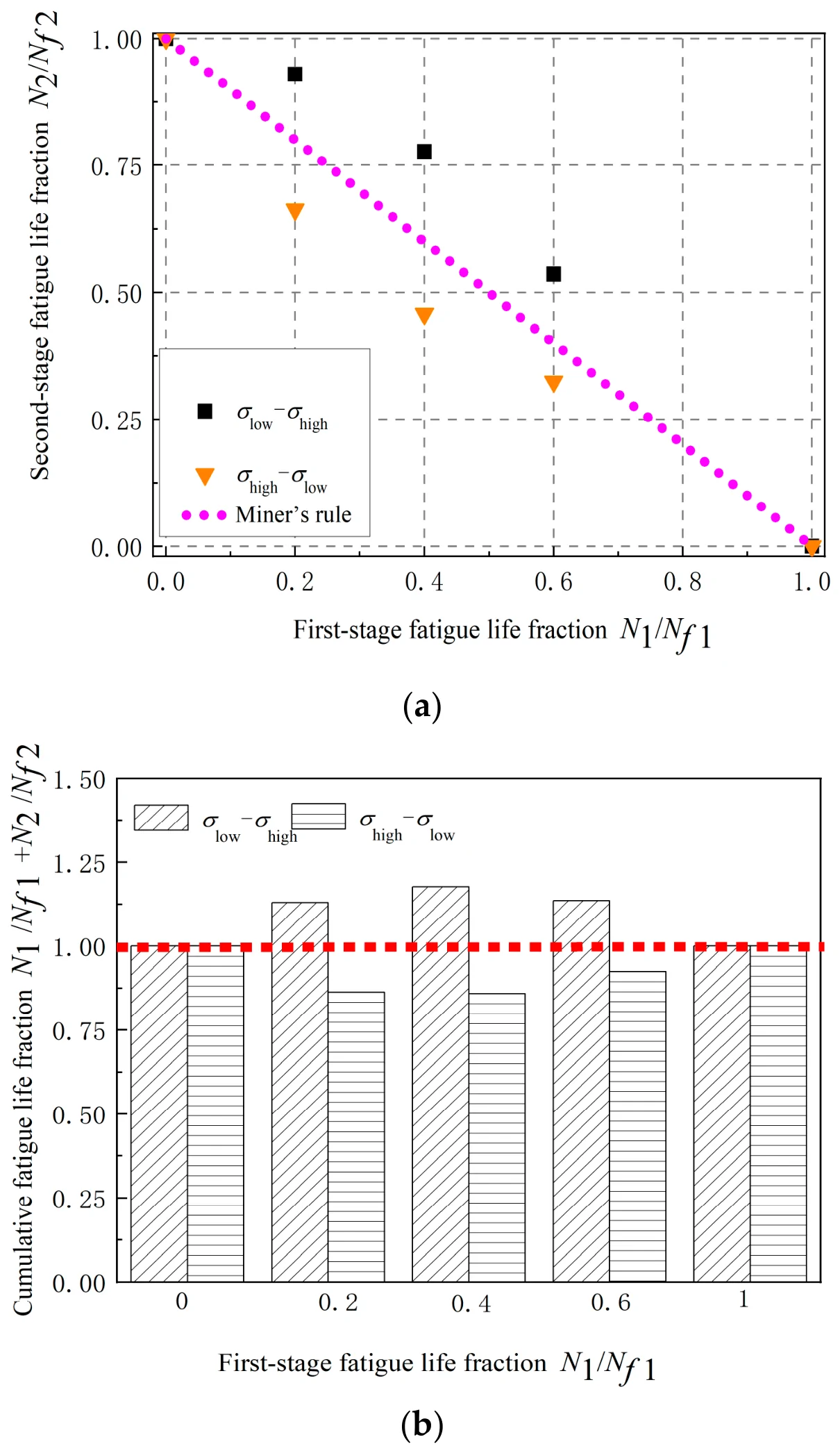
Life fractions of SBS-modified asphalt mixtures under variable-stress-amplitude cyclic loading. (**a**) Second fatigue life fraction of SBS-modified asphalt mixtures. (**b**) Cumulative fatigue life fraction of SBS-modified asphalt mixtures.

When *N*_1_/*N_f_*_1_ + *N*_2_/*N_f_*_2_ does not equal 1, the corresponding fatigue damage accumulation is nonlinear. Figure 5 presents the fatigue life fraction results for SBS-modified asphalt mixtures under high–low and low–high stress sequences. The dashed line in Figure 5 represents Miner’s rule, where the cumulative fatigue life fraction equals 1, indicating linear damage accumulation. However, as shown in Figure 5, the fatigue life fraction data points for the SBS-modified asphalt mixtures do not coincide with this dashed line.

As the first fatigue life fraction increases gradually from 0 to 1, the second fatigue life fraction exhibits a nonlinear decreasing trend from 1 to 0. The second fatigue life fraction results are distributed both above and below the dashed line, corresponding to the conditions of *N*_1_/*N_f_*_1_ + *N*_2_/*N_f_*_2_ > 1 and *N*_1_/*N_f_*_1_ + *N*_2_/*N_f_*_2_ < 1, respectively. The analysis indicates that SBS-modified asphalt mixtures present prominent nonlinear damage accumulation behavior when subjected to diverse variable-amplitude cyclic loading sequences, a feature that cannot be reproduced by the conventional Miner’s rule. Accordingly, a nonlinear fatigue damage accumulation model should be constructed to describe such nonlinear damage evolution for SBS-modified asphalt mixtures under variable-amplitude cyclic stress conditions.

### 2.2. Investigation on Nonlinear Fatigue Damage Accumulation of SBS-Modified Asphalt Mixtures with Physical Gel Structures Considering Loading Sequence–Interaction Coupling Effects

#### 2.2.1. Characterization Analysis of Loading Sequence Effect on Fatigue Damage Accumulation of SBS-Modified Asphalt Mixtures

**Figure 6:**
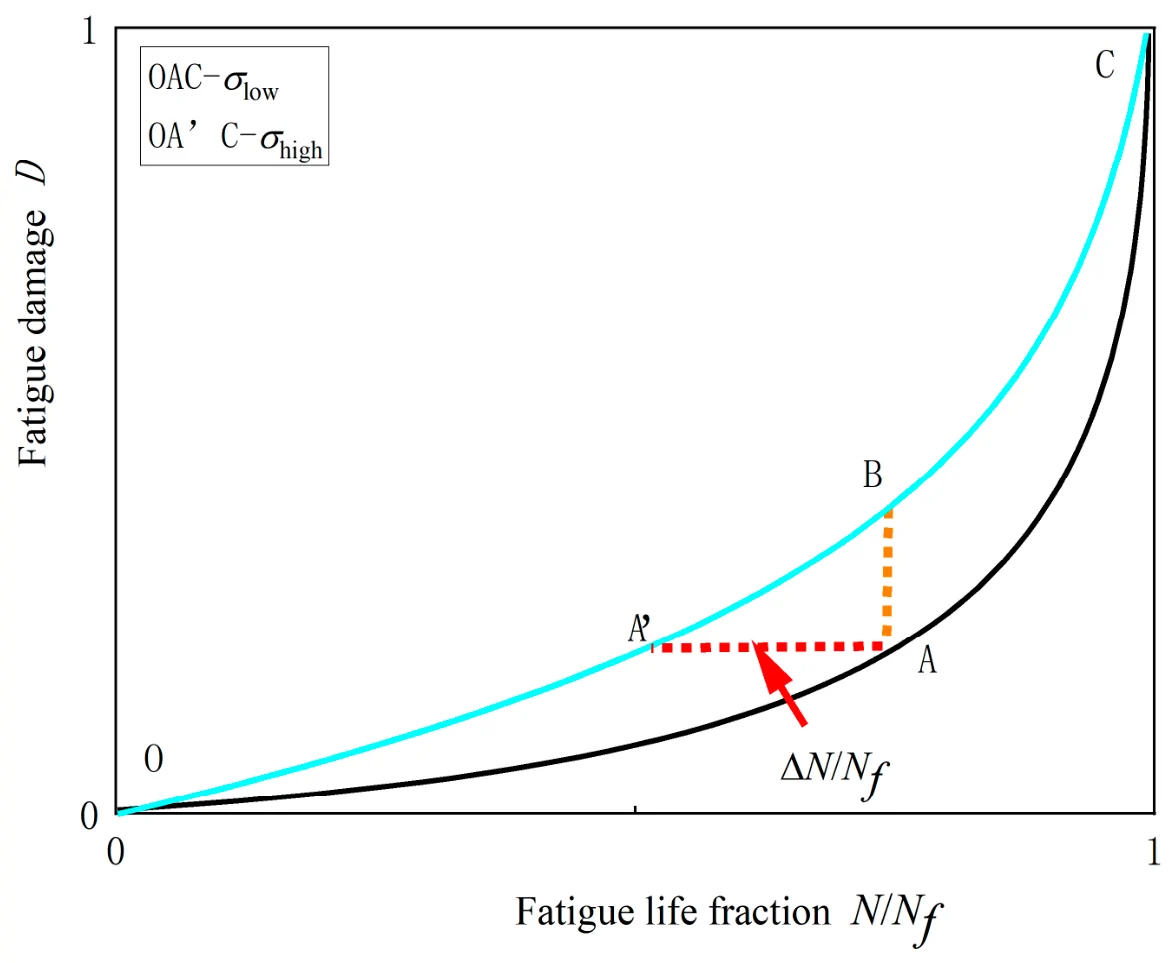
Schematic diagram of fatigue damage accumulation paths under variable-amplitude repeated loading.

Figure 6 presents a schematic diagram of the damage accumulation paths for SBS-modified asphalt mixtures under the loading sequences *σ*_high_–*σ*_low_ and *σ*_low_–*σ*_high_. Theoretically, the paths OAC and OA′C in Figure 6 represent the fatigue damage accumulation paths corresponding to *σ*_low_ and *σ*_high_, respectively, under constant-amplitude indirect repeated tensile loading.

When the loading sequence is *σ*_low_–*σ*_high_, the damage accumulation path during the first-stage loading with *N*_1_ cycles at *σ*_low_ is denoted as OA. Based on the equivalence of fatigue damage states, the damage induced by loading at *σ*_low_ up to point *A* can be considered equivalent to that induced by loading at *σ*_high_ up to point *A*′. The subsequent damage accumulation paths are then A′B and BC. Therefore, the overall damage accumulation path under the *σ*_low_–*σ*_high_ sequence is OA-A′C. It is worth noting that this equivalence process results in an overlap of A′A units along the horizontal direction between paths OA and A′B, where the fatigue life fraction corresponding to A′A is Δ*N*/*N_f_*. Consequently, the cumulative fatigue life fraction is not equal to 1, but rather equals 1 + Δ*N*/*N_f_*, i.e., *N*_1_/*N_f_*_1_ + *N*_2_/*N_f_*_2_ > 1. Conversely, when the loading sequence is *σ*_high_–*σ*_low_, the cumulative paths OA′ and AC lack A′A units along the horizontal direction, leading to *N*_1_/*N_f_*_1_ + *N*_2_/*N_f_*_2_ < 1. These findings indicate that the *σ*_high_–*σ*_low_ and *σ*_low_–*σ*_high_ sequences accelerate and decelerate, respectively, the accumulation of fatigue damage in SBS-modified asphalt mixtures, resulting in cumulative fatigue life fractions of less than 1 and greater than 1, respectively.

In the axle-load spectrum, the loading history induced by a preceding cyclic load can affect the subsequent fatigue damage accumulation caused by another cyclic load. This phenomenon is referred to as the loading interaction effect. During the first-stage loading, the fatigue damage induced by *N*_1_ cycles at the first-stage load amplitude *σ*_1_ is given by:

(5) $$D_{1}=1\;-{\left(1-\;\frac{N_{1}}{N_{f1}}\right)}^{\frac{1}{1+2\beta_{1}}}$$

where *D*_1_ is the fatigue damage induced during the first-stage loading, and *β*_1_ is the damage model parameter associated with the load amplitude *σ*_1_.

The fatigue damage state corresponding to *σ*_1_ during the first-stage loading is expressed as

(6) $$S_{\mathrm{fd}1}=S_{\mathrm{fd}}(\sigma_{1},\;\frac{N_{1}}{N_{f1}},1\;-{\left(1-\;\frac{N_{1}}{N_{f1}}\right)}^{\frac{1}{1+2\beta_{1}}})\;$$

When the load amplitude during the first-stage loading changes from *σ*_1_ to *σ*_2_, the corresponding fatigue damage state then becomes:

(7) $$S_{\mathrm{fd}1}=S_{\mathrm{fd}}(\sigma_{2},\;\frac{{N_{2}}^{\prime }}{N_{f2}},1\;-{\left(1-\;\frac{{N_{2}}^{\prime }\;}{N_{f2}}\right)}^{\frac{1}{1+2\beta_{2}}})\;$$

where *β*_2_ is the model parameter associated with the load amplitude *σ*_2_, and *N*_2_′ is the number of loading cycles required for *σ*_2_ to induce the same damage *D*_1_.

Based on the equivalence of fatigue damage states, the following relationship holds:

(8) $$\begin{array}{l} S_{\mathrm{fd}}(\sigma_{1},\;\frac{N_{1}}{N_{f1}},1\;-{\left(1-\;\frac{N_{1}}{N_{f1}}\right)}^{\frac{1}{1+2\beta_{1}}})\;=S_{\mathrm{fd}}(\sigma_{2},\;\frac{{N_{2}}^{\prime }}{N_{f2}},1\;-{\left(1-\;\frac{{N_{2}}^{\prime }}{N_{f2}}\right)}^{\frac{1}{1+2\beta_{2}}})\;\\ \;\;\;\;\;\;\;\;\;\;\;\;\;\;\;\;\;\;\;\;\;\;\;\;\;\;\;\;\Rightarrow \frac{{N_{2}}^{\prime }}{N_{f2}}=1-{\left(1-\frac{N_{1}}{N_{f1}}\right)}^{\frac{1+2\beta_{2}}{1+2\beta_{1}}} \end{array}$$

After converting the load amplitude during the first-stage loading from *σ*_1_ to *σ*_2_ through equivalence, the two-stage variable-amplitude cyclic loading can be treated as a constant-amplitude indirect repeated tensile loading at the load amplitude *σ*_2_. Since the two-stage variable-amplitude fatigue tests satisfy *N*_2_′ + *N*_2_ = *N_f_*_2_, it follows that:

(9) $$\frac{N_{2}}{N_{f2}}+\frac{{N_{2}}^{\prime }}{N_{f2}}=1$$

By defining the loading sequence factor, the fatigue damage accumulation model considering the effect of loading sequence is obtained, as presented below:

(10) $$\;\frac{N_{1}}{N_{f1}}+{\left(\frac{N_{2}}{N_{f2}}\right)}^{{\lambda_{1,2}}^{-1}}=1$$

where *β*_1_ is the model parameter for *σ*_1_ during the first stage, and *β*_2_ is the model parameter for *σ*_2_ during the second stage.

When the first-stage variable-amplitude cyclic load equals the second-stage load amplitude, the loading sequence factor is equal to 1. In this case, fatigue damage accumulates linearly, and the cumulative fatigue life fraction equals 1. When the variable-amplitude loading sequence is *σ*_low_–*σ*_high_, the loading sequence factor is $\lambda_{1,2}=\frac{1+2\beta_{\mathrm{low}}}{1+2\beta_{\mathrm{high}}}<1$, resulting in a cumulative fatigue life fraction greater than 1. Similarly, when the sequence is *σ*_high_–*σ*_low_, the loading sequence factor is $\lambda_{1,2}=\frac{1+2\beta_{\mathrm{high}}}{1+2\beta_{\mathrm{low}}}>1$, resulting in a cumulative fatigue life fraction of less than 1. The above analysis indicates that the accumulation of fatigue damage in SBS-modified asphalt mixtures is significantly dependent on the loading sequence, and the sequence factor *β* can be used to characterize this dependence

#### 2.2.2. Development of a Nonlinear Fatigue Damage Accumulation Model Considering Loading Sequence–Interaction Coupling Effects

For the classical two-level variable-amplitude cyclic fatigue test, the loading procedure is as follows: the first-stage load amplitude (*σ*_1_) is applied for *N*_1_ cycles, followed by the second-stage load amplitude (*σ*_2_) applied for *N*_2_ cycles until fatigue failure occurs. When considering only the effect of loading sequence, the fatigue damage state induced by *σ*_1_ and *σ*_2_ can be equivalently represented by that induced by *σ*_2_ applied for *N*_2_ + *N*_2_′ cycles. The fatigue damage induced by *σ*_2_ is $D=f(\sigma_{2},\;\frac{N}{N_{f2}})$, and the corresponding fatigue damage state is expressed as

(11) $$S_{\mathrm{fd}2}=S_{\mathrm{fd}}(\sigma_{2},\;\frac{{N_{2}}^{\prime }}{N_{f2}}+\frac{N_{2}}{N_{f2}},f(\sigma_{2},\;\frac{{N_{2}}^{\prime }}{N_{f2}}+\frac{N_{2}}{N_{f2}}))$$

However, due to the loading interaction effect, the loading history induced by *σ*_1_ can influence the fatigue damage accumulation process under *σ*_2_, causing the fatigue damage induced by *σ*_2_ to change from $D=f(\sigma_{2},\;\frac{N}{N_{f2}})$ to $D=f^{\prime }(\sigma_{2},\;\frac{N}{N_{f2}})$. Meanwhile, the number of loading cycles required for σ_2_ to induce the same damage *D*_1_ will change from *N*_2_′ to *N*_2_″, as expressed by

(12) $$D_{1}=f(\sigma_{1},\;\frac{N}{N_{f1}})=f^{\prime }(\sigma_{2},\;\frac{{N_{2}}^{\prime \prime }}{N_{f2}})$$

Accordingly, the fatigue damage state induced by *σ*_1_ and *σ*_2_ can be equivalently represented by that induced by *σ*_2_ applied for *N*_2_ + *N*_2_″ cycles, with the corresponding fatigue damage state expressed as

(13) $$\begin{array}{l} S_{\mathrm{fd}2}=S_{\mathrm{fd}}(\sigma_{1},\;\frac{N_{1}}{N_{f1}},f(\sigma_{1},\;\frac{N_{1}}{N_{f1}}))+S_{\mathrm{fd}}(\sigma_{2},\;\frac{N_{2}}{N_{f2}},f(\sigma_{2},\;\frac{N_{2}}{N_{f2}}))\\ \;\;\;\;\;\;=S_{\mathrm{fd}}(\sigma_{2},\;\frac{{N_{2}}^{\prime \prime }}{N_{f2}}+\frac{N_{2}}{N_{f2}},f^{\prime }(\sigma_{2},\;\frac{{N_{2}}^{\prime \prime }}{N_{f2}}+\frac{N_{2}}{N_{f2}})) \end{array}$$

Due to the loading interaction effect, the equivalent number of loading cycles changes from *N*_2_′ to *N*_2_″. The greater the difference between *σ*_1_ and *σ*_2_, the more pronounced the loading interaction effect becomes, and the larger the discrepancy between *N*_2_′ and *N*_2_″. The ratio of *σ*_1_ to *σ*_2_ can therefore be used to characterize the influence of loading interaction on fatigue damage accumulation.

In this study, the fatigue damage model $D=1\;-{\left(1-\;\frac{N}{N_{f}}\right)}^{\frac{1}{1+2\beta }}$ is employed to describe the evolution of fatigue damage in SBS-modified asphalt mixtures. Based on the structural characteristics of this damage model, $D=f^{\prime }(\sigma_{2},\;\frac{N}{N_{f2}})$ can be determined. It is assumed that:

(14) $$f^{\prime }(\sigma_{2},\;\frac{{N_{2}}^{\prime \prime }}{N_{f2}})=1\;-{\left[{\left(1-\;\frac{{N_{2}}^{\prime \prime }}{N_{f2}}\right)}^{\frac{1}{1+2\beta_{2}}}\right]}^{\frac{\sigma_{1}\gamma_{1,2}}{\sigma_{2}}}$$

By combining Equations (13) and (14), the following relationship is obtained:

(15) $$\begin{array}{l} f(\sigma_{1},\;\frac{N_{1}}{N_{f1}})=f^{\prime }(\sigma_{2},\;\frac{{N_{2}}^{\prime \prime }}{N_{f2}})=1\;-{\left(1-\;\frac{N_{1}}{N_{f1}}\right)}^{\frac{1}{1+2\beta_{1}}}=1\;-{\left[{\left(1-\;\frac{{N_{2}}^{\prime \prime }}{N_{f2}}\right)}^{\frac{1}{1+2\beta_{2}}}\right]}^{\frac{\sigma_{1}\gamma_{1,2}}{\sigma_{2}}} \end{array}$$

The $\lambda_{1,2}=\frac{1+2\beta_{2}}{1+2\beta_{1}}$, $\omega_{\;1,2}=\frac{\sigma_{2}}{\sigma_{1}\gamma_{1,2}}$, and $\kappa_{\;1,2}=\lambda_{\;1,2}\omega_{\;1,2}$ are defined. *σ*_1_ and *σ*_2_ are the stress amplitudes expressed in the same unit (kPa in this study), ensuring that *ω*_1,2_ is dimensionless and independent of the choice of a consistent unit system. The nonlinear fatigue damage accumulation model considering both loading sequence and interaction effects is obtained as follows:

(16) $$\frac{N_{1}}{N_{f1}}+{\left(\frac{N_{2}}{N_{f2}}\right)}^{{\kappa_{1,2}}^{-1}}=1$$

where *λ*_1,2_ denotes the loading sequence factor that quantifies how loading sequence influences fatigue damage accumulation in SBS-modified asphalt mixtures. *ω*_1,2_ represents the loading interaction factor for describing the impact of loading interaction on damage evolution. *γ*_1,2_ stands for the model parameter used to quantify loading interaction effects. Furthermore, *κ*_1,2_ is defined as the nonlinear fatigue damage accumulation factor, accounting for the joint influences of loading sequence and loading interaction on damage evolution. A larger absolute deviation between *κ*_1,2_ and unity corresponds to stronger nonlinear behavior in fatigue damage accumulation. When *κ*_1,2_ = 1, this model degenerates into Miner’s linear damage accumulation rule.

In an *n*-level variable-amplitude cyclic fatigue test, the loading procedure is as follows: stress levels *σ*_1_, *σ*_2_, *σ*_3_, …, *σ*_n_ are applied for *N*_1_, *N*_2_, *N*_3_, …, *N_n_* cycles, respectively, until fatigue failure occurs. The corresponding nonlinear fatigue damage accumulation model is given by:

(17) $$\begin{array}{l} \frac{N_{1}}{N_{f1}}+{\left\{\frac{N_{2}}{N_{f2}}+{\left[\frac{N_{3}}{N_{f3}}+\ldots \ldots +{\left(\frac{N_{n}}{N_{fn}}\right)}^{\frac{1+2\beta_{n-1}}{1+2\beta_{n}}\frac{\sigma_{n-1}\gamma_{n-1,n}}{\sigma_{n}}}\right]}^{\frac{1+2\beta_{2}}{1+2\beta_{3}}\frac{\sigma_{2}\gamma_{2,3}}{\sigma_{3}}}\right\}}^{\frac{1+2\beta_{1}}{1+2\beta_{2}}\frac{\sigma_{1}\gamma_{1,2}}{\sigma_{2}}}=1\\ \;\;\;\;\;\;\;\;\Rightarrow \frac{N_{1}}{N_{f1}}+{\left\{\frac{N_{2}}{N_{f2}},+,{\left[\frac{N_{3}}{N_{f3}}+\ldots \ldots +{\left(\frac{N_{n}}{N_{fn}}\right)}^{{\kappa_{n-1,n}}^{-1}}\right]}^{{\kappa_{2,3}}^{-1}}\right\}}^{{\kappa_{1,2}}^{-1}}=1 \end{array}$$

where *N_f_*_n_ denotes the fatigue life corresponding to *σ*_n_, while *κ_n__−_*_1__,n_ is the nonlinear fatigue damage accumulation factor during the *n*-th stage of variable-amplitude cyclic loading. When *κ*_1,2_ = *κ*_2,3_ = … = *κ_n__−_*_1_*_,n_* = 1, the model reduces to Miner’s linear damage accumulation rule.

#### 2.2.3. Analysis of Second Fatigue Life Fraction

**Figure 7:**
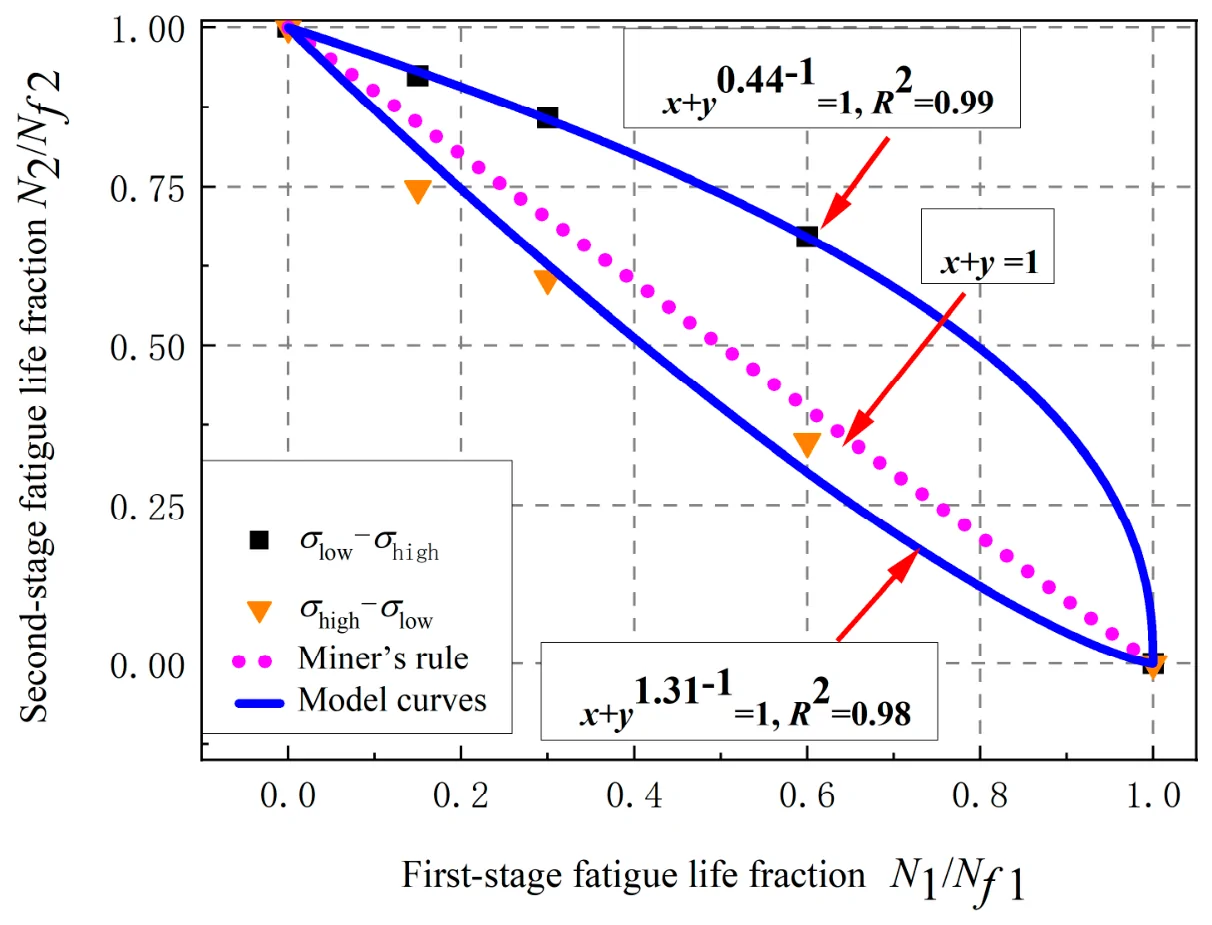
Fitting results of nonlinear fatigue damage accumulation model of asphalt mixtures.

The proposed nonlinear fatigue damage accumulation model was employed to fit the fatigue life fraction results for SBS-modified asphalt mixtures under both *σ*_high_–*σ*_low_ and *σ*_low_–*σ*_high_ loading sequences, with the fitting results presented in Figure 7 and Table 2. As shown in Figure 7 and Table 2, the established model accurately describes the nonlinear accumulation of fatigue damage in SBS-modified asphalt mixtures under variable-amplitude cyclic loading.

The dashed line in Figure 7 corresponds to Miner’s rule, where the cumulative fatigue life fraction equals 1. As shown in Figure 7, under the *σ*_low_–*σ*_high_ loading sequence, *κ* is less than 1, and the corresponding second fatigue life fraction data points lie above the dashed line, indicating that the *σ*_low_–*σ*_high_ sequence retards the accumulation of fatigue damage. When the loading sequence is reversed to *σ*_high_–*σ*_low_, *κ* exceeds 1, and the data points fall below the dashed line, suggesting that the *σ*_high_–*σ*_low_ sequence accelerates fatigue damage accumulation. It can reasonably be inferred that for newly constructed asphalt pavements, allowing light vehicles to operate for a certain period before introducing heavy vehicles may slow the accumulation of fatigue damage, thereby extending the pavement’s service life.

To further verify the effectiveness of the developed model, additional variable-amplitude fatigue tests were conducted under the conditions listed in Table 3, with other test parameters held constant. As reported in previous studies [20,21], a characterization approach for nonlinear fatigue damage accumulation was developed to consider loading sequence effects yet ignore loading interaction effects, and the corresponding model is given in Equation (10). For comparison purposes, the conventional Miner’s rule, the nonlinear model considering only sequence effects, and the model developed herein are designated as Model 1, Model 2, and Model 3, respectively. A comparative analysis of the second fatigue life fraction predictions from the three models is presented in Table 3. As shown in Table 3, the relative error of Model 1 is significantly larger than that of Models 2 and 3, which is attributed to the fact that Miner’s rule can only describe linear fatigue damage accumulation. Overall, the relative error of Model 2 is greater than that of Model 3, because Model 2 considers only the loading sequence effect. Theoretically, when the loading sequence is the dominant factor affecting damage accumulation and the interaction effect is secondary, Model 2 may outperform Model 3.

The above analysis indicates that, compared with Model 1, both Model 2 and Model 3 provide more accurate predictions because they account for nonlinear damage accumulation. In most cases, Model 3 exhibits smaller relative errors than Model 2, demonstrating the benefit of incorporating loading interaction effects. However, in the specific case of the *σ*_low_–*σ*_high_ sequence, Model 3 shows a slightly larger error (12.38%) than Model 2 (9.40%), suggesting that when the loading sequence effect is dominant and the interaction effect is secondary, the additional interaction term may not always yield improvement. This observation highlights the need for further investigation into the conditions under which loading interaction effects become pronounced.

### 2.3. Fatigue Life Prediction of SBS-Modified Asphalt Mixtures with Physical Gel Structures Under Variable-Amplitude Indirect Repeated Tensile Loading

By introducing a nonlinear fatigue damage accumulation factor, the traditional linear fatigue equation is modified, based on which a fatigue life prediction model for SBS-modified asphalt mixtures considering the loading sequence–interaction coupling effects is established.

#### 2.3.1. Analysis of Variable-Amplitude Repeated Loading Effect on Fatigue Life

**Figure 8:**
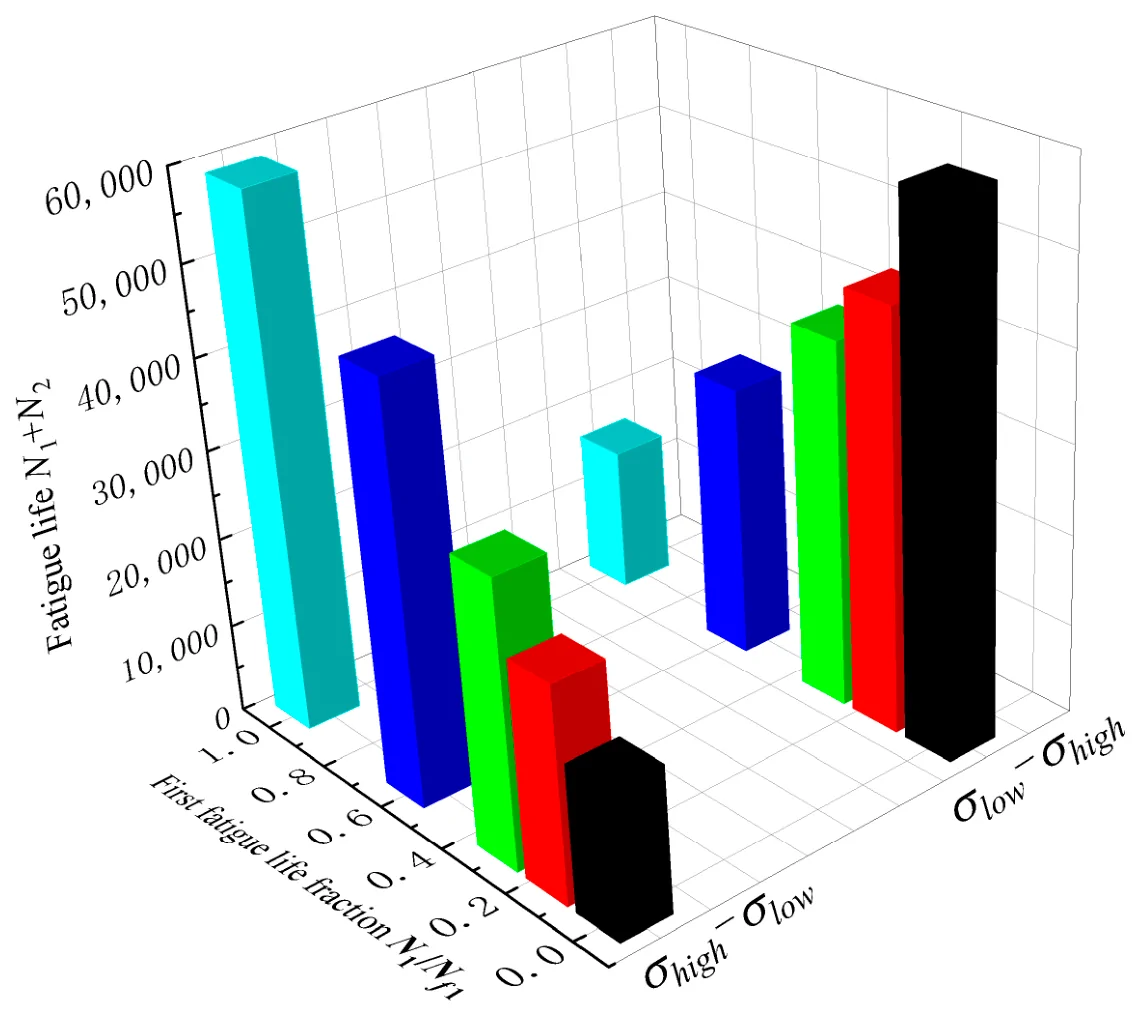
Fatigue life of variable-amplitude indirect repeated tensile loading tests.

The fatigue life results of SBS-modified asphalt mixtures under different loading modes are presented in Figure 8. As shown in Figure 8, under the *σ*_high_–*σ*_low_ loading mode, the fatigue life exhibits a decreasing trend with increasing first fatigue life fraction. This is because SBS-modified asphalt mixtures exhibit relatively slow damage accumulation at *σ*_low_, whereas the introduction of *σ*_high_ accelerates damage accumulation, leading to increased damage per unit cycle.

As *N*_1_/*N_f_*_1_ increases from 0 to 1, the duration of the rapid damage accumulation phase is extended, while the number of cycles required to reach the same damage level decreases, resulting in a reduction in fatigue life. When the loading mode is switched from *σ*_high_–*σ*_low_ to *σ*_low_–*σ*_high_, as shown in Figure 8, the fatigue life exhibits a monotonic increase with increasing first fatigue life fraction. This is because the introduction of *σ*_low_ retards the damage accumulation process, thereby reducing the damage accumulation rate. As the first fatigue life fraction increases, the duration of the slow accumulation phase lengthens, and more cycles are required to accumulate the same damage level, leading to an increase in fatigue life.

The above analysis indicates that variable-amplitude cyclic loading can significantly affect the fatigue life of SBS-modified asphalt mixtures by either accelerating or retarding the accumulation of fatigue damage. Therefore, there is a need to develop a fatigue life prediction model applicable to SBS-modified asphalt mixtures subjected to variable-amplitude indirect repeated tensile loading.

#### 2.3.2. Analysis of Fatigue Life Prediction Models Under Constant-Amplitude Indirect Repeated Tensile Loading

By integrating damage evolution equation $\frac{dD}{dN}={\left[1-{\left(1-D\right)}^{1+\beta }\right]}^{\alpha }{\left[\frac{\sigma }{M\left(1-D\right)}\right]}^{\beta }$ derived from irreversible thermodynamics theory, the relationship for SBS-modified asphalt mixtures under constant-amplitude indirect repeated tensile loading can be derived as follows:

(18) $$N_{f}=\frac{1}{\left(1+\beta )(1-\alpha \right)}{\left(\frac{\sigma }{M}\right)}^{-\beta }$$

By taking the logarithm of both sides of the above equation, the relationship between fatigue life *N*_f_ and load amplitude *σ* on a logarithmic scale is obtained as follows:

(19) $$\mathrm{lg}N_{f}=\mathrm{lg}\frac{1}{\left(1+\beta )(1-\alpha \right)}+\beta \mathrm{lg}(2\beta )-\beta \mathrm{lg}\sigma$$

Similarly, based on the damage evolution equation $\frac{dD}{dN}=(\sigma^{2}M{)}^{\beta }{\left(1-D\right)}^{-2\beta }$ derived from viscoelastic damage mechanics theory, the following relationship can be obtained:

(20) $$\mathrm{lg}N_{f}=\mathrm{lg}\frac{M^{-\beta }}{1+2\beta }-2\beta \mathrm{lg}\sigma$$

Based on the above analysis, the traditional linear fatigue equation under constant-amplitude indirect repeated tensile loading can be derived as follows:

(21) $$\mathrm{lg}N_{f}=a+b\mathrm{lg}\sigma$$

where a and b are model parameters, and *σ* is the stress amplitude in kPa.

By fitting the fatigue test results under constant-amplitude indirect repeated tensile loading with Equation (21), the traditional linear fatigue equation for SBS-modified asphalt mixtures under such loading conditions is obtained, as presented in Equation (22). The standard errors for *a* and *b* are 0.423 and 0.156, respectively, with corresponding 95% confidence intervals of (13.402, 15.093) and (−4.768, −4.144).

lg*N_f_* = 14.2476 − 4.456lg*σ*, *R*^2^ = 0.957 (22)

#### 2.3.3. Development of a Fatigue Life Prediction Model Considering Loading Sequence–Interaction Coupling Effects

This study adopts the nonlinear fatigue damage accumulation theory. Based on the equivalence of fatigue damage states, the established nonlinear damage accumulation model is given by Equation (17). From this equation, the fatigue life fraction model under variable-amplitude indirect repeated tensile loading can be derived as follows:

(23) $$\begin{array}{l} \frac{N_{n}}{N_{fn}}={\left\{{\left[{\left(1-\;\frac{N_{1}}{N_{f1}}\right)}^{\frac{1+2\beta_{2}}{1+2\beta_{1}}\frac{\sigma_{2}}{\sigma_{1}\gamma_{1,2}}}\;-\frac{N_{2}}{N_{f2}}\right]}^{\frac{1+2\beta_{3}}{1+2\beta_{2}}\frac{\sigma_{3}}{\sigma_{2}\gamma_{2,3}}}\ldots \ldots -\frac{N_{n-1}}{N_{fn-1}}\right\}}^{\frac{1+2\beta_{n}}{1+2\beta_{n-1}}\frac{\sigma_{n}}{\sigma_{n-1}\gamma_{n-1,n}}}\\ \;\;\;\;\;\;\;={\left\{{\left[{\left(1-\;\frac{N_{1}}{N_{f1}}\right)}^{\kappa_{1,2}}\;\;-\frac{N_{2}}{N_{f2}}\right]}^{\kappa_{2,3}}\ldots \ldots -\frac{N_{n-1}}{N_{fn-1}}\right\}}^{\kappa_{n,n-1}} \end{array}$$

Accordingly, the fatigue life prediction model considering both loading sequence and interaction effects is obtained as follows:

(24) Nf=∑i=1i=n−110^(a+blgσi)NiNfi+10^(a+blgσn)(1− N1Nf1)κ1,2  −N2Nf2κ2,3……−Nn−1Nfn−1κn,n−1

where *κ_n_*_,*n*−1_ is the nonlinear fatigue damage accumulation factor. When *κ_n_*_,*n*−1_ = 1, the fatigue life prediction model does not account for the effects of loading sequence and loading interaction on fatigue life.

Next, taking the three-level variable-amplitude cyclic loading fatigue test as an example, the proposed nonlinear fatigue life prediction model is analyzed. When *n* = *3*, the third fatigue life fraction is given by:

(25) $$\begin{array}{l} \frac{N_{3}}{N_{f3}}={\left[{\left(1-\frac{N_{1}}{N_{f1}}\right)}^{\frac{1+2\beta_{2}}{1+2\beta_{1}}\frac{\sigma_{2}}{\sigma_{1}\gamma_{1,2}}}-\frac{N_{2}}{N_{f2}}\right]}^{\frac{1+2\beta_{3}}{1+2\beta_{2}}\frac{\sigma_{3}}{\sigma_{2}\gamma_{2,3}}}={\left[{\left(1-\frac{N_{1}}{N_{f1}}\right)}^{\lambda_{1,2}\omega_{1,2}}-\frac{N_{2}}{N_{f2}}\right]}^{\lambda_{2,3}\omega_{2,3}}\\ \;\;\;\;\;\;\;={\left[{\left(1-\frac{N_{1}}{N_{f1}}\right)}^{\kappa_{1,2}}-\frac{N_{2}}{N_{f2}}\right]}^{\kappa_{2,3}} \end{array}$$

Accordingly, the nonlinear fatigue life prediction model under three-level variable-amplitude cyclic loading is obtained as follows:

(26) Nf=10^(a+blgσ1)N1Nf1+10^(a+blgσ2)N2Nf2        +10^(a+blgσ3)(1− N1Nf1)κ12  −N2Nf2κ23

**Figure 9:**
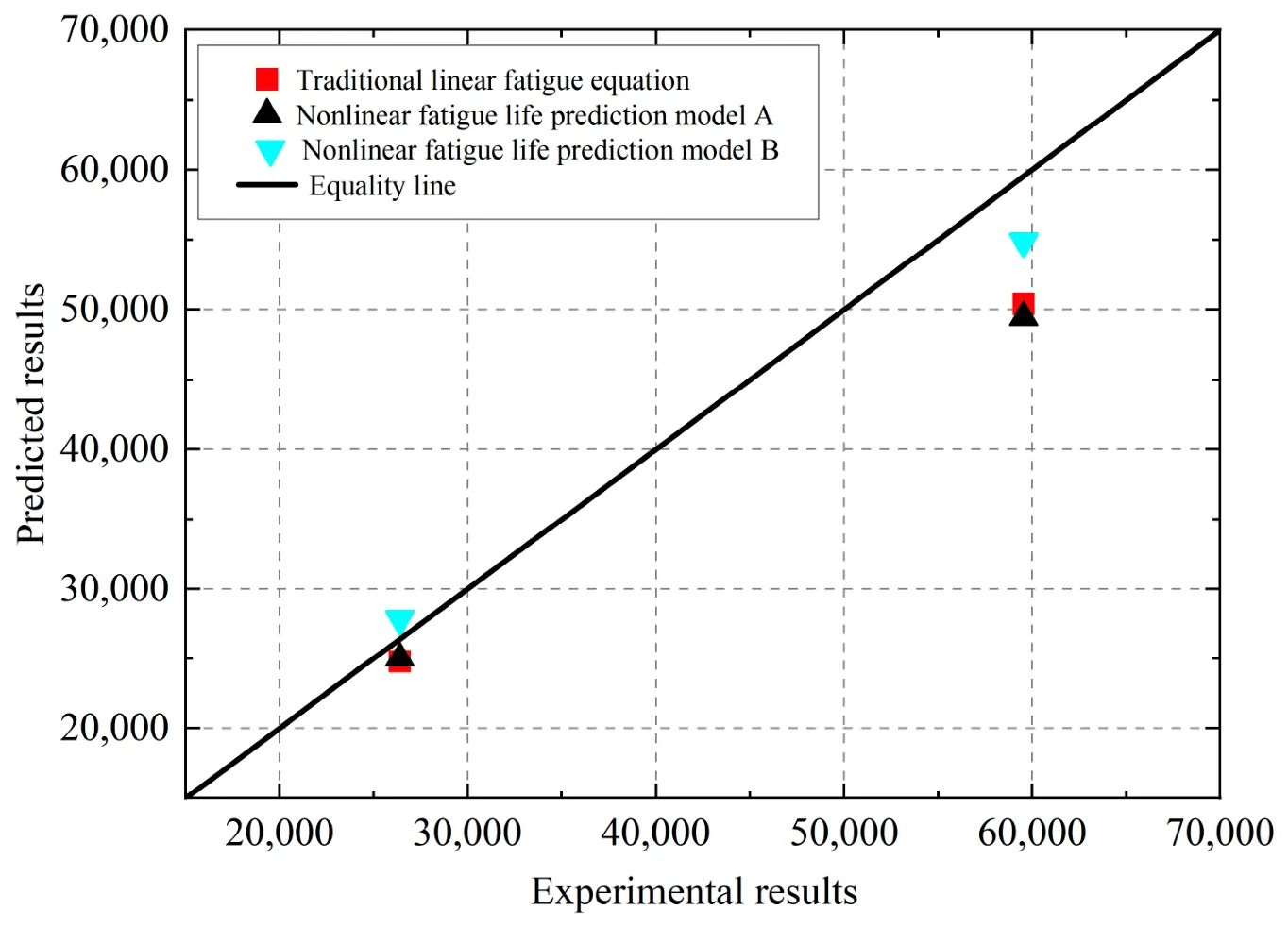
Predicted results of fatigue life.

To further validate the effectiveness of the proposed model, an additional three-level variable-amplitude cyclic loading fatigue test was conducted under the conditions listed in Table 4, with the loading sequences of *σ*_low_–*σ*_high_–*σ*_low_ and *σ*_high_–*σ*_low_–*σ*_high_ selected while keeping other test parameters unchanged. When the interaction factor is set to 1, the nonlinear fatigue life prediction model that accounts only for loading sequence is obtained, denoted as Nonlinear Model A. The model established in this study is denoted as Nonlinear Model B. The traditional linear fatigue equation, Nonlinear Model A, and Nonlinear Model B were, respectively, employed to predict the fatigue life, with the prediction results presented in Figure 9 and Table 5. The diagonal line in Figure 9 represents perfect agreement between the predicted and experimental results; data points above the diagonal indicate overestimation, while those below indicate underestimation.

As shown in Figure 9 and Table 5, although the predicted data points do not perfectly coincide with the 45° line, they are generally distributed around it, indicating that all three fatigue life prediction methods can predict the fatigue life of SBS-modified asphalt mixtures with reasonable accuracy. Although the traditional linear fatigue equation provides reasonably accurate predictions, it fails to account for the effects of loading sequence–interaction. Compared with the traditional linear fatigue equation and Nonlinear Model A, the proposed Nonlinear Model B can simultaneously account for the effects of loading sequence–interaction on the fatigue life of SBS-modified asphalt mixtures and, in most cases, yields more accurate predictions. However, given the limited number of three-level loading histories examined (two sequences), the observed improvements should be interpreted as preliminary evidence rather than conclusive proof of general superiority. Further validation with a broader range of loading sequences and stress levels is warranted. Theoretically, when the loading sequence is the dominant factor affecting fatigue life and loading interaction is a secondary factor, Nonlinear Model A may yield more accurate predictions than Nonlinear Model B.

## 3. Conclusions

(1) The *σ*_low_–*σ*_high_ and *σ*_high_–*σ*_low_ loading sequences retard and accelerate, respectively, the fatigue damage accumulation process of SBS-modified asphalt mixtures. The cumulative fatigue life fractions under the *σ*_low_–*σ*_high_ and *σ*_high_–*σ*_low_ sequences are greater than 1 and less than 1, respectively.

(2) Loading interaction affects the fatigue damage accumulation of SBS-modified asphalt mixtures, and the loading interaction factor can be used to characterize the dependence of damage accumulation on loading interaction.

(3) By introducing the nonlinear fatigue damage accumulation factor into the traditional Miner’s rule, a nonlinear fatigue damage accumulation model considering the loading sequence–interaction coupling effects can be established. This model can accurately trace the nonlinear fatigue damage accumulation of SBS-modified asphalt mixtures.

(4) A nonlinear fatigue life prediction model considering the loading sequence–interaction coupling effects can be established by introducing a nonlinear fatigue damage accumulation factor into the traditional linear fatigue equation. This model can predict the fatigue life of SBS-modified asphalt mixtures under variable-amplitude cyclic loading with reasonable accuracy.

The present model, though effective for two-level variable-amplitude loading, has not yet been extended to general random loading conditions—a critical step for practical implementation. Future research should address this gap by developing a generalized formulation that accounts for stochastic stress histories and multi-factor interactions. Additionally, the model’s robustness should be tested across different material systems and environmental regimes, and its predictions should be benchmarked against field data from in-service pavements.

## 4. Materials and Methods

### 4.1. Test Materials

In this paper, the selected commercial SBS-modified asphalt with physical gel structures (Zhengzhou, China) has an SBS modifier content of 4.5%, and its basic technical properties are shown in Table 6. The Superpave performance grade of the SBS-modified asphalt is 76-16 (PG76-16). The selected aggregate is granite (Luoyang, China), and the gradation type is stone mastic asphalt (SMA), with the relevant parameters given in Table 7.

### 4.2. Preparation of SBS-Modified Asphalt Mixtures with Physical Gel Structures

**Figure 10:**
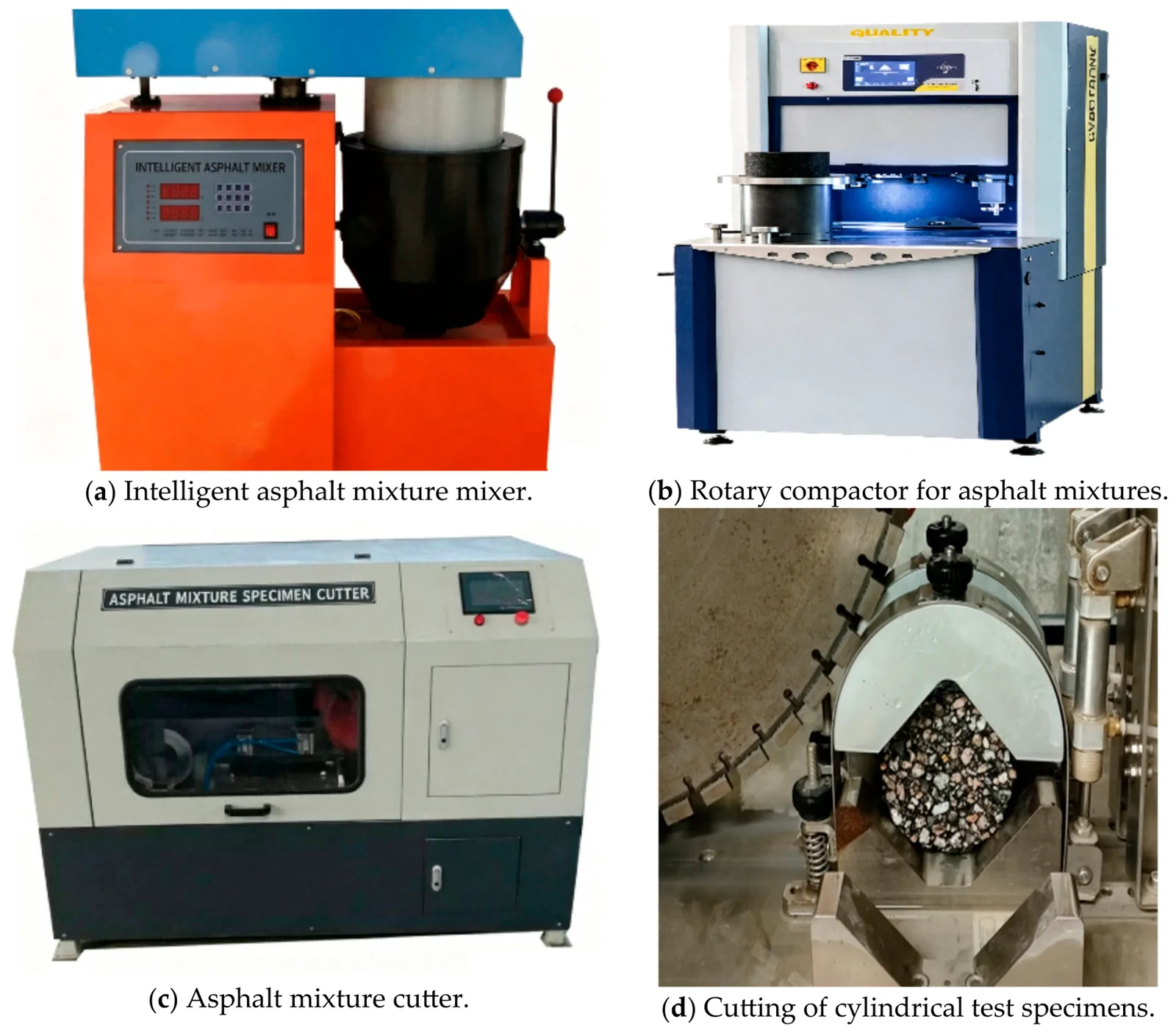
Molding and cutting of test specimens.

The mineral aggregates and asphalt were accurately weighed according to the designed asphalt content of 6%. The aggregates were preheated to 175 °C and then placed into the intelligent asphalt mixture mixer shown in Figure 10a, where they were mechanically stirred at 175 ± 5 °C for approximately 90 s to ensure uniform coating of the aggregates by the asphalt. The prepared mixture was then loaded into a 100 mm diameter metal mold and placed in the gyratory compactor shown in Figure 10b. A vertical pressure of 600 kPa and a rotation speed of 30 rpm were applied for compaction. The number of compaction cycles was adjusted to achieve a final specimen height of 150 mm, with the air void content strictly controlled within 4% ± 0.5% through real-time volumetric monitoring.

The specimens were cooled with an electric fan for 30 min prior to demolding. Both ends of each specimen were then precisely trimmed using a specialized double-face cutting machine shown in Figure 10c, with approximately 15 mm removed from each end. The cut surfaces were kept flat and perpendicular to the specimen axis, as depicted in Figure 10d. Cylindrical specimens with a diameter of 100 mm and a height of 40 mm were finally obtained for subsequent mechanical performance testing. Throughout the entire preparation process, particular attention was paid to temperature control, compaction effort, and dimensional accuracy to ensure uniform and dense internal structures within the specimens.

### 4.3. Test Methods

**Figure 11:**
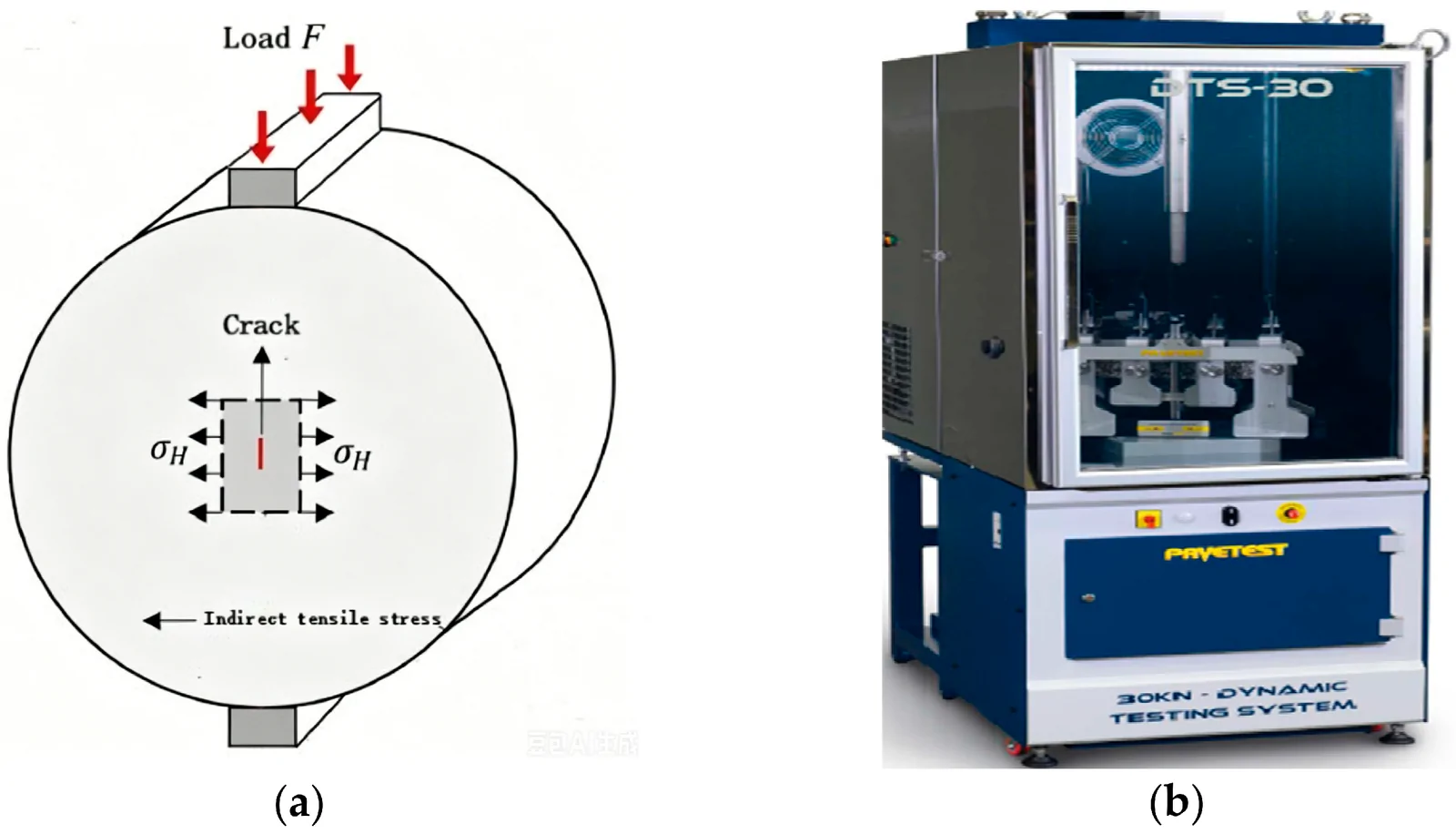
Schematic diagram of an indirect tensile fatigue test. (**a**) Schematic diagram of mechanical response under indirect repeated tensile loading. (**b**) Dynamic test system loading equipment.

The indirect repeated tensile loading test has gained broad acceptance in pavement practice, primarily owing to its straightforward sample fabrication, high test repeatability, and operational ease in the field. These attributes make it a suitable choice for investigating the rate-dependent fatigue damage of SBS-modified asphalt mixture. Hence, it was adopted in this study to investigate variable-amplitude fatigue life prediction for SBS-modified asphalt mixtures. A dynamic testing system, as illustrated in Figure 11b, was used as the loading device. A loading frequency of 10 Hz, corresponding to a vehicle speed of 60–65 km/h, was selected, and the test temperature was set to 20 °C, a moderate temperature prone to fatigue cracking. Five replicate tests were conducted for each loading condition.

#### 4.3.1. Indirect Uniaxial Tensile Loading Test

To determine the load amplitude for the constant-amplitude indirect repeated tensile loading tests, indirect uniaxial tensile loading tests were first carried out. The loading mode was displacement-controlled at a constant rate of 50 mm/min. The dynamic testing system was activated to apply axial pressure to the specimen until fatigue fracture occurred, and the peak load (*F*_max_) was recorded. The tensile stress was calculated approximately using Equation (27). The indirect uniaxial tensile stress corresponding to *F*_max_ is defined as the strength (*σ*_max_), which was determined to be 895 kPa for the SBS-modified asphalt mixture specimens used in this study.

(27) $$\sigma =\frac{2F}{\pi \;t\;\Omega }$$

where *F* is the applied force (N), t is the specimen height (mm), and *Ω* is the specimen diameter (mm).

#### 4.3.2. Constant-Amplitude Indirect Repeated Tensile Loading Test

**Figure 12:**
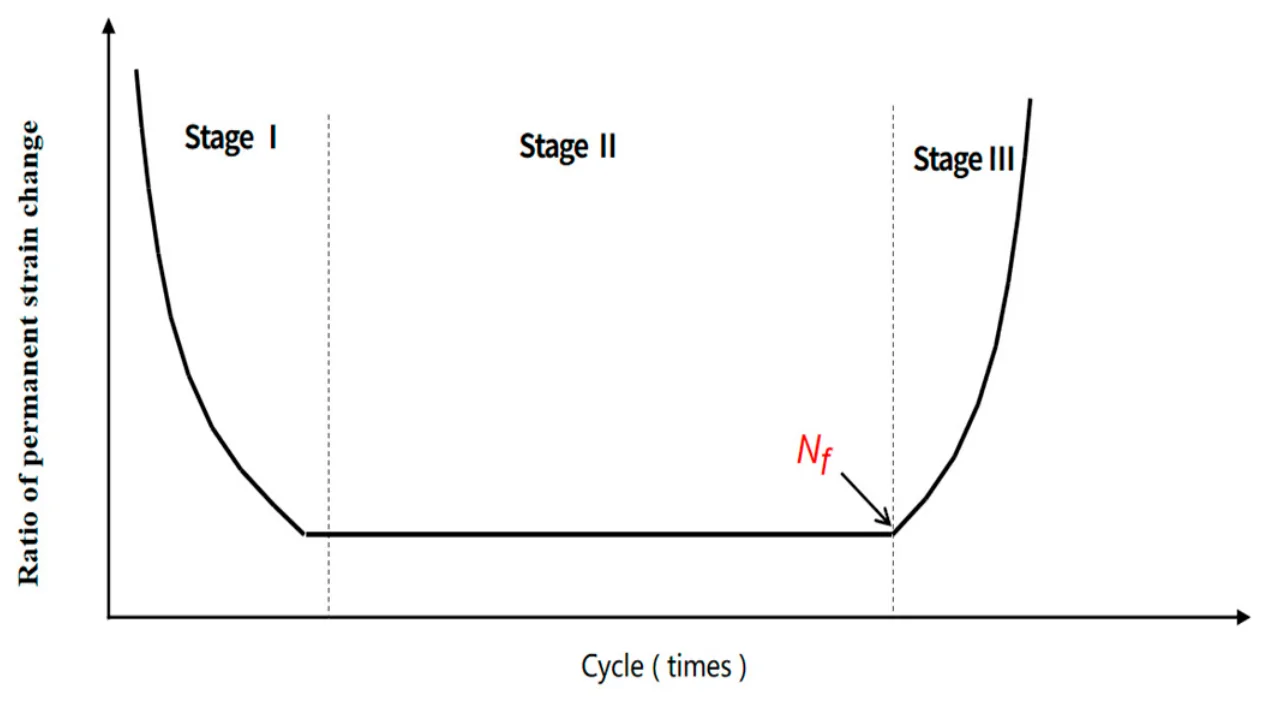
Typical curve of the ratio of permanent strain change.

The loading amplitudes were set at 0.15*σ*_max_ and 0.2*σ*_max_, corresponding to the high load amplitude (*σ*_high_) and low load amplitude (*σ*_low_), respectively, to conduct constant-amplitude indirect repeated tensile loading tests to determine the constant-amplitude fatigue life of SBS-modified asphalt mixtures. The tests were performed under stress-controlled mode. The transverse tensile strain at the valley of each loading cycle was taken as the permanent strain (*PS*). The ratio of permanent strain change (*RPSC*) was calculated using Equation (28), and a typical *RPSC* curve is shown in Figure 12. The cycle number corresponding to the transition point between the second and third stages of the *RPSC* curve was the fatigue life [21]. The constant-amplitude fatigue life of SBS-modified asphalt mixtures is presented in Table 8.

(28) $$RPSC=\frac{PS_{N+1}-PS_{N}}{PS_{N}}$$

where *PS*_N_ is the permanent strain at the *N*-th cycle.

#### 4.3.3. Variable-Amplitude Indirect Repeated Tensile Loading Test

**Figure 13:**
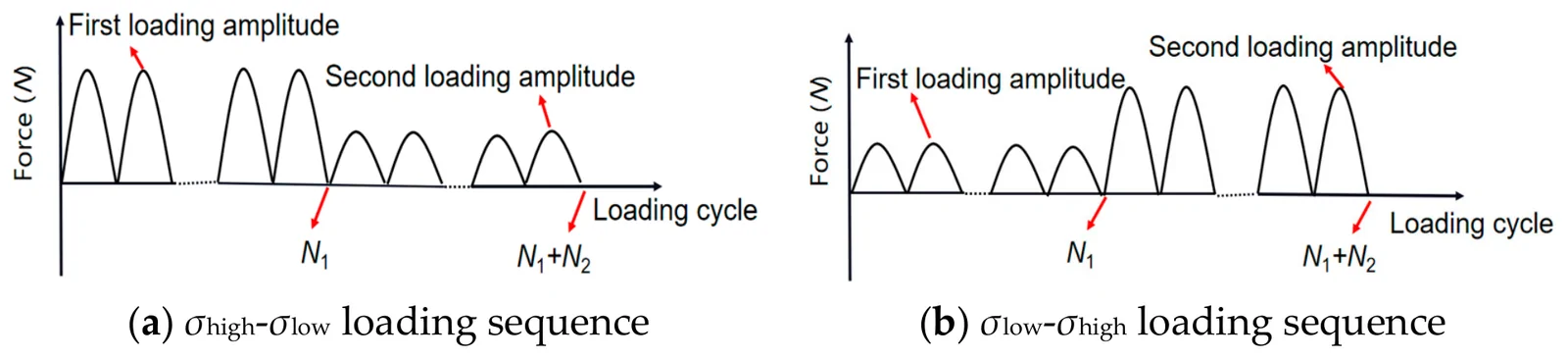
Loading sequences of variable-amplitude indirect repeated tensile loading test.

The loading waveform and frequency used in the variable-amplitude indirect repeated tensile loading tests were kept consistent with those in the constant-amplitude tests. Two loading sequences, namely high–low (*σ*_high_–*σ*_low_) and low–high (*σ*_low_–*σ*_high_), as illustrated in Figure 13, were selected for the variable-amplitude tests. These sequences correspond to the traffic scenarios of “heavy vehicles first, followed by light vehicles” and “light vehicles first, followed by heavy vehicles,” respectively. The loading procedure was as follows: (1) the first-stage load amplitude was applied for *N*_1_ cycles; (2) the second-stage load amplitude was then applied for *N*_2_ cycles until fatigue failure occurred. The cumulative fatigue life results of the tests are presented in Table 9.

In this study, *N*_1_/*N_f_*_1_, *N*_2_/*N_f_*_2_, *N*_1_/*N_f_*_1_ + *N*_2_/*N_f_*_2_, and *N*_1_ + *N*_2_ are defined as the first-stage life fraction, second-stage life fraction, cumulative life fraction, and cumulative fatigue life, respectively. Here, *N_f_*_1_ and *N_f_*_2_ are the constant-amplitude fatigue lives corresponding to the first- and second-stage load amplitudes, respectively. The values of *N*_1_/*N*_f1_ were set to 0, 0.15, 0.3, 0.6, and 1. For the *σ*_high_–*σ*_low_ loading sequence, the first- and second-stage load amplitudes are *σ*_high_ and *σ*_low_, respectively; for the *σ*_low_–*σ*_high_ sequence, they are *σ*_low_ and *σ*_high_, respectively.

## Figures and Tables

**Table 1:** Fitting results of the permanent strain model incorporating damage effects.

Loading Amplitude	Parameter	Estimate	Standard Error	95% CI	*R* ^2^
*σ*_low_	*α*	5.18 × 10^−6^	1.20 × 10^−7^	(4.94 × 10^−6^, 5.42 × 10^−6^)	0.987
	*ρ*	−7.23 × 10^−10^	2.10 × 10^−11^	(−7.65 × 10^−10^, −6.81 × 10^−10^)	
	*γ*	5.83 × 10^−14^	1.50 × 10^−15^	(5.53 × 10^−14^, 6.13 × 10^−14^)	
	*λ*	0.001	5.20 × 10^−5^	(0.00090, 0.00110)	
	*κ*	0.00686	0.00031	(0.00624, 0.00748)	
	*β*	8.70471	0.423	(7.858, 9.551)	
*σ*_high_	*α*	8.90 × 10^−6^	2.30 × 10^−7^	(8.44 × 10^−6^, 9.36 × 10^−6^)	0.971
	*ρ*	−1.88 × 10^−09^	5.60 × 10^−11^	(−1.99 × 10^−9^, −1.77 × 10^−9^)	
	*γ*	2.29 × 10^−13^	7.80 × 10^−15^	(2.13 × 10^−13^, 2.45 × 10^−13^)	
	*λ*	0.00112	6.80 × 10^−5^	(0.00098, 0.00126)	
	*κ*	0.0104	0.00052	(0.00936, 0.01144)	
	*β*	7.39884	0.385	(6.629, 8.169)	

**Table 2:** Fitting results of nonlinear fatigue damage accumulation model.

Loading Sequence	Parameter	Estimate	Standard Error	95% CI	*R* ^2^
*σ*_low_–*σ*_high_	*λ*_1,2_	0.858130	0.042	(0.774, 0.942)	0.99
	*ω*_1,2_	0.508641	0.031	(0.447, 0.570)	
	*κ*_1,2_	0.44	0.038	(0.364, 0.516)	
*σ*_high_–*σ*_low_	*λ*_1,2_	1.165324	0.063	(1.039, 1.292)	0.98
	*ω*_1,2_	1.123764	0.058	(1.008, 1.240)	
	*κ*_1,2_	1.31	0.071	(1.168, 1.452)	

**Table 3:** Predicted fatigue life results for second fractions.

Materials	Loading Sequence	*N*_1_/*N_f_*_1_	Experimental Value of *N*_2_/*N_f_*_2_	Coefficient of Variation (%)	Model 1		Model 2		Model 3	
					Predicted Value of *N*_2_/*N_f_*_2_	Relative Error (%)	Predicted Value of *N*_2_/*N_f_*_2_	Relative Error (%)	Predicted Value of *N*_2_/*N_f_*_2_	Relative Error (%)
SBS-Modified Asphalt Mixture	*σ*_low_–*σ*_high_	0.4	0.712	9.789	0.6	15.730	0.645	9.397	0.800	12.380
	*σ*_high_–*σ*_low_	0.4	0.482	10.196	0.6	24.481	0.551	14.400	0.512	6.275

**Table 4:** Test results for third fatigue life fractions.

Materials	Loading Sequence	*N*_1_/*N_f_*_1_	*N*_2_/*N_f_*_2_	*N*_3_/*N_f_*_3_	Standard Deviation	Standard Error	Coefficient of Variation (%)
SBS Modified Asphalt Mixture	*σ*_low_–*σ*_high_–*σ*_low_	0.3	0.2	0.655	0.110	0.049	16.774
	*σ*_high_–*σ*_low_–*σ*_high_	0.3	0.2	0.600	0.091	0.041	15.172

**Table 5:** Predicted results of fatigue life.

Loading Sequence	Experimental *N_f_*	Traditional Model		Model A		Model B	
		Predicted *N_f_*	Relative Error (%)	Predicted *N_f_*	Relative Error (%)	Predicted *N_f_*	Relative Error (%)
*σ*_low_–*σ*_high_–*σ*_low_	59,550	50,410	15.35	49,457	16.949	54,863	7.871
*σ*_high_–*σ*_low_–*σ*_high_	26,393	24,773	6.138	24,992	5.309	27,851	5.524

**Table 6:** Technical properties of SBS asphalt binder.

Indicator	SBS Modified Asphalt
Penetration (25 °C) (0.1 mm)	57
Kinematic viscosity (135 °C) (Pa·s)	1.54
Flash Point (°C)	308
Solubility (%)	96.83
Softening Point (°C)	54.9
Ductility (5 °C, 5 cm/min) (cm)	33.4
Elastic Recovery (25 °C) (%)	87

**Table 7:** Aggregate gradation.

Composition	Relative Density (g/cm^3^)	Dimensions (mm)	Percentage (%)
Coarse Aggregate	2.642	14–10	3.5
	2.663	10–5	59.5
	2.709	5–2.36	9.0
Fine Aggregate	2.649	2.36–0.075	16.5
Filler	2.661	<0.075	9.5
Lime	2.587		2.0

**Table 8:** Fatigue life under constant-amplitude indirect repeated tensile loading.

Loading Amplitude	Loading Amplitude	Mean Value	Standard Deviation	Standard Error	Coefficient of Variation (%)
*σ*_low_	134 kPa	58,955	4468	1999	7.579
*σ*_high_	179 kPa	16,228	1304	583	8.036

**Table 9:** Fatigue life under variable-amplitude indirect repeated tensile loading.

Loading 1	*N*_1_/*N*_f1_	*N*_1_ + *N*_2_	Standard Deviation	Standard Error	Coefficient of Variation/%
*σ*_low_–*σ*_high_	0	16,228	1304	583	8.036
	0.15	23,842	2523	1128	10.582
	0.3	31,607	2829	1265	8.952
	0.6	46,265	4578	2048	9.896
	1	58,955	4468	1998	7.579
*σ*_high_–*σ*_low_	0	58,955	4468	1998	7.579
	0.15	46,491	4312	1928	9.275
	0.3	40,485	4336	1939	10.710
	0.6	30,291	3002	1342	9.909
	1	16,228	1304	583	8.036

## Data Availability

Data are contained within the article. Some or all of the data, models, or code that support the findings of this study are available from the corresponding author upon reasonable request.
